# Association between season of vaccination and antibody levels against infectious diseases

**DOI:** 10.1017/S0950268820002691

**Published:** 2020-11-05

**Authors:** T. C. Abreu, H. Boshuizen, L. Mollema, G. A. M. Berbers, H. Korthals Altes

**Affiliations:** 1 Centre for Infectious Disease Control, National Institute for Public Health and the Environment (RIVM), Bilthoven, the Netherlands; 2 Centre for Nutrition, Prevention and Health Services, National Institute for Public Health and the Environment (RIVM), Bilthoven, the Netherlands

**Keywords:** Childhood vaccination schedule, seasonality, serology, vaccine

## Abstract

Vaccination has reduced the disease burden of vaccine-preventable diseases. However, the extent to which seasonal cycles of immunity could influence vaccine-induced immunity is not well understood. A national cross-sectional serosurveillance study performed in the Netherlands (Pienter-2) yielded data to investigate whether season of vaccination was associated with antibody responses induced by DT-IPV (diphtheria, tetanus and poliomyelitis), MMR (measles, mumps and rubella) and meningococcus C (MenC) vaccines in children. In total, 434 children met the inclusion criteria to study DT-IPV immunity, 811 for MMR and 311 for MenC. Differences in log(antibody levels) by season of vaccination were investigated with linear multivariable regression analyses. Seroconversion rates varied according to season of vaccination for rubella (90% of autumn-vaccinated children *vs.* 99% of winter-vaccinated had concentrations above cut-off levels). Summer-vaccinated boys showed a slower decline of tetanus antibodies (6% per month), in comparison with winter-vaccinated boys. In conclusion, season of vaccination showed little association with immunological protection. However, a number of associations were seen with a *P*-value of about 0.03; and adding data from a just-completed nationwide serological study might add more power to the current study. Further immunological and longitudinal investigations could help understand the mechanisms of seasonal influence in vaccine-induced responses.

## Introduction

Over the last century, vaccination successfully reduced the disease burden of the more severe vaccine-preventable diseases (VPDs), such as diphtheria, pertussis, tetanus and poliomyelitis [1]. In the Netherlands, the majority of vaccines offered by the National Immunization Programme (NIP) cover more than 90% of the target population (children aged 0–19 years), following an age-based administration schedule [2].

The magnitude of antibody levels following vaccination varies between persons due to several factors: gender [3], age at vaccine inoculation [4], pre-existing antibody levels [5], health status [6] and immune system cycles [7].

Seasonal patterns in immunity, for example, cytokine profiles [8], or gene expression patterns [9], could affect the quality of the response to a vaccine. Through competition or enhancement, arms of the immune response influence each other in a possibly seasonal way. Seasonal differences in vaccine response may be explained by: variation in dose or duration of UV exposure, which may alter immune system and host resistance [10]; higher infection rates in certain seasons [11], which could possibly ‘prime’ the immune system producing an altered vaccine-induced response. Factors probably less important in the Netherlands are reduction in food availability, which impacts maternal and child's nutritional status and child's vaccine response [12], and pronounced differences in average temperatures throughout the year that may impact the vaccine cold chain and possibly vaccine response [13].

Past studies focused on single pathogens, had short follow-up and were mostly carried out in non-temperate climate zones. Two studies in temperate climate reported no association: in the Netherlands, immune responses to hepatitis B vaccine in college students were not associated with season of vaccination, nor were rubella and measles antibody levels following vaccination in children [14]. Inconclusive seasonal patterns were reported for immune response to hepatitis B vaccine in Austria [15].

The current exploratory study aims to investigate whether season of vaccination is associated with strength of the antibody response against a selection of pathogens in a representative nationwide sample of children from the Netherlands.

## Methods

### Study design and setting

Serology was obtained from the Pienter-2 study, a national cross-sectional serosurveillance study performed between February 2006 and December 2007 in the Netherlands. In brief, Pienter-2 assessed the population's immune status against VPDs covered by the NIP through blood samples and self-administered questionnaires. Dutch inhabitants from 0 to 79 years old were included in the survey in two samples. A nationwide sample of 6348 participants, including an oversample of non-western migrants (*n* = 646), and a sample of 1518 participants from low vaccination coverage (LVC) areas (1517). A full description can be found elsewhere [16, 17].

### Selection of vaccine-preventable diseases

We included a number of VPDs covered by the Dutch NIP in 2006/2007: diphtheria, tetanus and poliomyelitis, covered by the combination vaccine DP(a)T-IPV-Hib; measles, mumps and rubella, covered by the MMR combination vaccine; and meningococcal C disease, covered by the monovalent MenC vaccine.

Four VPDs covered by the NIP were not included: pertussis (due to the replacement of whole cell vaccine by acellular vaccine within the study period) [18]; pneumococcal disease (due to insufficient data, since the vaccine was implemented in 2006) [19]; *Haemophilus influenzae* type b (due to an increase in vaccine failure in 2002) [20] and hepatitis B (since it was only offered to risk groups at the time of study) [21].

### Selection of age ranges and vaccines

**Fig. 1.**
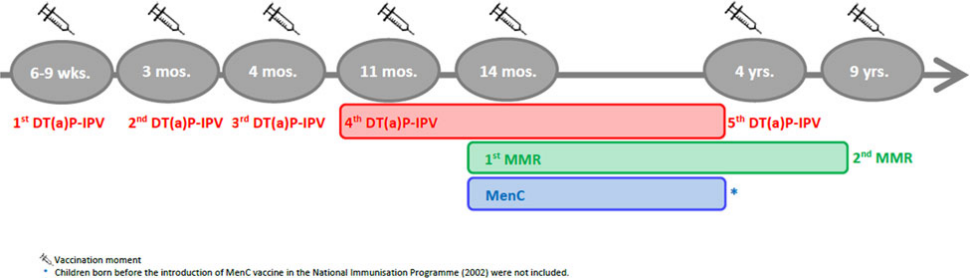
Timeline of the vaccination schedule of the Dutch National Immunisation Programme for diphtheria, tetanus, poliomyelitis, measles, mumps, rubella and meningococcal C disease at the time of the Pienter-2 study (2006/2007). Coloured boxes indicate age range selected for the study of sub-samples.

**Fig. 2.**
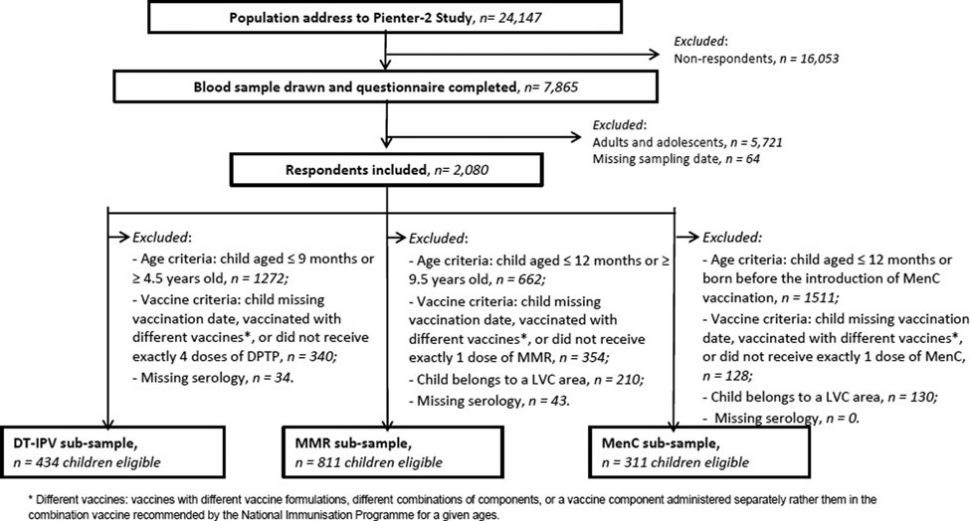
Flow chart of exclusion/inclusion criteria, Pienter-2 study.

According to the NIP schedule, vaccines are administered at distinct moments, either alone or in combination vaccines, and in a different number of doses (Fig. 1). Therefore, specific criteria applied to the selection of age ranges for the study of vaccine-induced immunity for each VPD (Fig. 2). When more than one vaccination was required to protect a child against a VPD, we considered the determining vaccination moment, the one in which the largest difference between pre- and post-vaccination antibody levels was observed (Guy Berbers, personal communication), as described in the literature [22]. Therefore, for DT-IPV, the season of the fourth vaccine dose given (at 11 months of age) was selected; for MMR, the season of the first vaccine dose (at 14 months of age) was considered.

The children in the study received the DT(a)P-IPV-Hib vaccine (Infanrix-IPV + Hib (GSK) or Infanrix Hexa (GSK) or Pediacel (SP-MDS)), the MMR vaccine (M-M-R VaxPro (SP-MDS)) and the MenC vaccine (NeisVac-C (Baxter)), according to the NIP schedule [2]. Vaccination dates were determined based on data from the nationwide information system Praeventis.

The concentrations of vaccine antibodies were assessed with the multiplex immunoassay (MIA). For diphtheria and tetanus, antibodies were determined by pentaplex MIA [23]. For poliomyelitis, anti-polio titres were measured via a standard neutralisation test [24]. For the simultaneous detection of antibodies against measles, mumps and rubella, a fluorescent bead-based MIA was performed [25]. Finally, the concentrations of MenC antibodies were determined using a fluorescent-bead-based MIA [26].

The cut-off values taken as seroconversion thresholds were: 0.1 IU/ml (full protection) and 0.01 IU/ml (basic immunity) for diphtheria [27], 0.1 IU/ml (full protection) and 0.01 IU/ml (basic immunity) for tetanus [28], 1:8 for the three polio types [29, 30], 0.2 IU/ml for measles [31, 32], 45 RU/ml for mumps [33], 10 IU/ml rubella [33] and 2 μg/ml for MenC [34–36] (Supplementary Table S1).

### Selection of sub-samples

In the current study, the exclusion criteria (Fig. 2) for all respondents were: missing information on sampling date, missing serology data and being born before 1996 (i.e. adolescent or adult at study sampling period). Along with exclusion due to missing vaccination date, additional specific exclusion criteria applied to each (combination) vaccine, therefore three sub-samples were created: DT-IPV, MMR and MenC, with possible multiple-occurrence of participants across sub-samples.

For the DT-IPV sub-sample, we excluded children who had not received exactly four DT(a)P-IPV-Hib vaccine doses (recommended, but not necessarily at 2, 3, 4 and 11 months of age). Children who had not received four vaccine doses or were younger than 9 months old and children who had received the booster dose at about 4 years of age or were older than 4.5 years old, were also excluded.

For the MMR sub-sample, we excluded children who had not received exactly one MMR vaccine dose (recommended, but not necessarily at 14 months of age); children receive a second dose MMR at 9 years of age, giving a relatively large time window for study.

For the MenC sub-sample, unvaccinated children, participants who were born before the MenC mass catch-up campaign (November 2002), and children with multiple MenC vaccinations were excluded.

In LVC areas, there was some circulation of MMR pathogens around or during the sampling period [37–39], and a relatively high incidence of meningococcal C disease before the introduction of the vaccine in 2002 [40]. We therefore excluded children from the LVC areas – potentially more exposed to the above-mentioned VPDs [41] – from the analyses.

### Selection of variables

#### Dependent and independent variables

Serum antibody levels (vaccine antigen-specific antibody) were measured following previously described laboratory methods for the Pienter-2 serosurveillance study [23–26]. Season of vaccination was defined as the quarter of the year in which the child was vaccinated (spring: April–June; summer: July–September; autumn: October–December and winter: January–March).

#### Covariates

The following covariates were included in the multivariable analysis: time post-vaccination, to account for possible waning antibody levels over time; and sex, considering sex-based differences in immune responses following vaccination [3]. Time post-vaccination was included as a continuous variable, measured as months between vaccination moment and serum sampling.

### Statistical analysis

Descriptive analyses were performed for population characteristics. Antibody levels were log-transformed or log 2-transformed (poliovirus antibody levels), to normalise the data. Geometric mean titres/concentrations (GMT/C) with 95% confidence intervals (95% CI) and seroconversion rates were calculated. Pairwise Pearson correlation between antibody levels against different pathogens within individuals was performed along with Bonferroni correction for multiple testing. Differences in log(antibody levels) between seasons of vaccination were tested with linear multivariable regression analysis, using ‘winter’ and ‘boy’ as reference levels for season of vaccination and sex, respectively. The percentage difference in antibody levels was obtained by subtracting 1 from the exponential of the regression coefficient *β* and multiplying it by 100: Difference = [(*e^β^* − 1) × 100].

Preliminary graphical analysis suggested sex differences in antibody levels according to season of vaccination and time post-vaccination (Supplementary Fig. S1). Therefore, the full model was applied, with a three-way interaction term (season of vaccination × sex × time post-vaccination), and all lower-order interaction terms and main effects, for each pathogen. Backward model selection was performed. If including three-way interaction terms improved the fit of the model, the analysis was stratified by sex to ease the interpretation. ‘Age at vaccination’ was not included in the model due to little variance and predictive power. The significance level was set at 0.01 rather than 0.05, to account for multiple testing.

R version 3.5.1 (R Development Core Team) and packages ‘lm’ and ‘lme4’ were used to analyse the data.

### Sensitivity analysis

In the Pienter-2 study, non-Western migrants are oversampled, and thus this group is overrepresented in our sub-samples. This group might have higher antibody levels [24], possibly due to genetic differences and/or re-exposure to pathogens during visits to the country of origin. Therefore, we performed a sensitivity analysis on the national sample only, excluding oversampled non-Western migrant children.

To account for possible non-linearity of log(antibody) decay over time – associated, for example, with a vaccine eliciting two pools of antibodies with different decay rates –, an alternative model replacing the variable time post-vaccination by (log)time post-vaccination was also fitted. Goodness-of-fit was compared with that of the final model selected for each VPD.

## Results

Of the 7865 respondents in the Pienter-2 study, covering all age groups, 2080 were children with information about sampling date (Fig. 2). After application of the sub-samples exclusion criteria, 434 children with a ‘follow-up’ period of about 45 months were eligible for the DT-IPV sub-sample, 811 for the MMR sub-sample with a ‘follow-up’ period of about 100 months and 311 for the MenC sub-sample with a ‘follow-up’ period of about 36 months (Figs 1 and 2). The sub-samples’ characteristics are presented in Table 1. Distribution of children per group (national sample, oversampled children with non-Western background and, for DT-IPV sub-sample only, individuals from LVC areas) did not differ across seasons (*P* > 0.1). Median age at vaccination and time post-vaccination were relatively consistent across seasons of vaccination for all sub-samples, except for time post-vaccination for the DT-IPV sub-sample (*P* *=* 0.018), which varied by as much as 8 months (summer *vs.* spring vaccination).

**Table 1.** Distribution of sample characteristics per season of vaccination, Pienter-2 study

Samples characteristics	Summer	Autumn	Winter	Spring	*P*-value
DT-IPV sub-sample (*n* = 434)	111 (25.6%)	106 (24.4%)	100 (23.0%)	117 (27.0%)	
Sex (female), *n* (%)	58 (52.2%)	51 (48.1%)	37 (37.0%)	51 (43.6%)	0.143^a^
Age at vaccination (months), median (IQR)	12 (11–12)	12 (11–12)	12 (11–12)	11 (11–12)	0.188^b^
Time post-vaccination (months), median (IQR)	21 (10–27)	19 (9–29)	18 (5–28)	13 (6–25)	0.018^b^
National sample, *n* (%)	75 (67.6%)	68 (64.1%)	76 (76.0%)	75 (64.1%)	0.228^a^
Belonging to a LVC area, *n* (%)	22 (19.8%)	29 (27.4%)	20 (20.0%)	30 (25.6%)	
Oversampled non-western migrant, *n* (%)	14 (12.6%)	9 (8.5%)	4 (4.0%)	12 (10.3%)	
MMR sub-sample (*n* = 811)	204 (25.2%)	242 (29.8%)	157 (19.4%)	208 (25.6%)	
Sex (female), *n* (%)	104 (50.9%)	117 (48.3%)	72 (45.9%)	92 (44.2%)	0.548^a^
Age at vaccination (months), median (IQR)	15 (14–16)	15 (14–16)	15 (14–16)	15 (14–15)	0.054^b^
Time post-vaccination (months), median (IQR)	46 (24–71)	41 (20–66)	39 (23–63)	38 (24–67)	0.169^b^
National sample, *n* (%)	151 (74.1%)	190 (78.5%)	115 (73.3%)	161 (77.4%)	0.541^a^
Oversampled non-western migrant, *n* (%)	53 (25.9%)	52 (21.5%)	42 (26.7%)	47 (22.6%)	
MenC sub-sample (*n* = 311)	67 (21.5%)	101 (32.5%)	57 (18.3%)	86 (27.7%)	
Sex (female), *n* (%)	31 (46.3%)	53 (52.5%)	27 (47.4%)	38 (44.2%)	0.705^a^
Age at vaccination (months), median (IQR)	14 (14–15)	15 (14–15)	15 (14–15)	15 (14–15)	0.300^b^
Time post-vaccination (months), median (IQR)	18 (8–22)	19 (8–25)	16 (9–24)	21 (11–27)	0.256^b^
National sample, *n* (%)	59 (88.1%)	83 (82.2%)	48 (84.2%)	76 (88.4%)	0.592^a^
Oversampled non-western migrant, *n* (%)	8 (11.9%)	18 (17.8%)	9 (15.8%)	10 (11.6%)	

IQR, interquartile range.

a Pearson Chi-square test.

b Kruskal–Wallis test.

### Geometric mean titre/concentration (GMT/C) and seroconversion rates

Table 2 shows the GMT/C (95% CI) for each pathogen, per season of vaccination and sex. Differences in GMT/C between boys and girls were statistically significant at *α* = 0.01 level for mumps and rubella. Seroconversion rates, applying the thresholds given for each pathogen (Supplementary Table S1) did not vary by season of vaccination, except for the rubella vaccine, where rates varied between 90% and 99% (*P* = 0.010), being lowest in children vaccinated in autumn and highest in winter.

**Table 2.** Geometric mean titres/concentrations (95% CI), per season of vaccination and sex, Pienter-2 study

	Winter	Spring	Summer	Autumn	*P*-value (overall mean sex difference)^a^
Diphtheria (IU/ml)	0.13 (0.09–0.19)	0.12 (0.09–0.17)	0.08 (0.06–0.10)	0.10 (0.08–0.13)	
Boys	0.14 (0.09–0.21)	0.13 (0.08–0.20)	0.08 (0.05–0.11)	0.09 (0.06–0.13)	0.848
Girls	0.12 (0.06–0.24)	0.12 (0.07–0.20)	0.08 (0.06–0.12)	0.12 (0.09–0.18)	
Tetanus (IU/ml)	0.83 (0.59–1.16)	0.91 (0.68–1.21)	0.63 (0.49–0.82)	0.80 (0.63–1.03)	
Boys	0.81 (0.52–1.26)	1.02 (0.69–1.50)	0.61 (0.44–0.85)	0.77 (0.55–1.08)	0.798
Girls	0.86 (0.49–1.51)	0.78 (0.50–1.22)	0.66 (0.45–0.97)	0.84 (0.57–1.24)	
Polio type 1 (log 2 GMT)	6.88 (6.29–7.47)	7.31 (6.74–7.87)	6.91 (6.42–5.93)	6.49 (5.95–7.03)	
Boys	6.83 (6.09–7.57)	7.32 (6.52–8.11)	6.25 (5.58–6.91)	5.62 (4.90–6.33)	0.068
Girls	6.97 (5.94–8.01)	7.29 (6.47–8.12)	6.59 (5.86– 7.32)	7.43 (6.68–8.19)	
Polio type 2 (log 2 GMT)	7.33 (6.70–7.96)	7.60 (7.01–8.19)	6.54 (5.99–7.09)	6.83 (6.26–7.40)	
Boys	7.06 (6.26–7.86)	7.79 (6.95–8.63)	6.08 (5.35–6.80)	5.98 (5.16–6.80)	0.034
Girls	7.78 (6.73–8.84)	7.35 (6.51–8.19)	6.97 (6.15–7.79)	7.75 (7.01–8.48)	
Polio type 3 (log 2 GMT)	6.04 (5.31–6.77)	6.43 (5.69–7.16)	5.05 (4.40–5.69)	5.56 (4.87–6.24)	
Boys	6.35 (5.43–7.27)	6.71 (5.67–7.75)	4.28 (3.45–5.12)	4.58 (3.68–5.48)	0.232
Girls	5.51 (4.25–6.77)	6.06 (5.01–7.11)	5.74 (4.79–6.70)	6.61 (5.61–7.60)	
Measles (IU/ml)	1.89 (1.56–2.29)	1.72 (1.48–2.01)	1.64 (1.42–1.89)	1.63 (1.40–1.90)	
Boys	1.89 (1.48–2.41)	1.58 (1.27–1.96)	1.54 (1.24–1.92)	1.60 (1.30–1.97)	0.279
Girls	1.88 (1.38–2.57)	1.93 (1.56–2.38)	1.74 (1.43–2.11)	1.66 (1.32–2.09)	
Mumps (RU/ml)	125 (102–152)	119 (103–137)	122 (107–139)	96 (80–116)	
Boys	132 (101–173)	97 (79–119)	104 (87–126)	88 (70–111)	0.009
Girls	117 (86–159)	154 (128–186)	142 (119–170)	106 (78–143)	
Rubella (IU/ml)	76 (65–89)	58 (50–68)	55 (47–65)	47 (39–56)	
Boys	76 (63–92)	48 (39–60)	46 (35–60)	39 (30–51)	0.001
Girls	76 (59–100)	73 (59–91)	66 (55–79)	56 (44–72)	
MenC (μg/ml)	0.66 (0.44–0.99)	0.80 (0.58–1.12)	0.77 (0.55–1.09)	0.95 (0.73–1.24)	
Boys	0.74 (0.48–1.15)	0.80 (0.51–1.24)	0.71 (0.44–1.16)	0.75 (0.54–1.04)	0.328
Girls	0.58 (0.28–1.21)	0.81 (0.49–1.36)	0.85 (0.51–1.42)	1.19 (0.80–1.77)	

*Note*: Confidence intervals (CI) in parentheses.

a *t* test for overall mean difference between boys and girls.

### Fitted regression

Season-stratified scatterplots of antibody levels against time post-vaccination and the fitted regression lines from the final models are displayed in Supplementary Figure S1. The model fit of tetanus and poliovirus serotype 1 was improved when a three-way interaction term was included, therefore the analyses for these pathogens were stratified by sex. The lines show a decline in antibody titres against diphtheria, tetanus, poliovirus and MenC with time since vaccination.

### Season of vaccination and antibody levels

No association between season of vaccination and antibody levels was found for any of the three poliovirus serotypes, for diphtheria, and for tetanus in girls (at *α* = 0.01 level, Table 3). In boys, the decline of tetanus antibodies after vaccination in summer was 6% per month slower than after vaccination in winter (Table 3: *β* = 0.06; *P* < 0.01; Supplementary Fig. S1B). This decline led antibody concentrations to levels close to sub-protective concentrations by 40 months post-vaccination (by the time children receive a booster against tetanus) (Supplementary Fig. S1B). No association was found between season of vaccination and antibody levels induced against measles, mumps and rubella (Table 4 and Supplementary Fig. S1F–H), nor meningococcus C (Table 5 and Supplementary Fig. S1I).

**Table 3.** Linear multivariate regression models estimates for antibody levels as a function of season of vaccination, time post-vaccination and sex for DT-IPV sub-sample, Pienter-2 study

	Diphtheria^a^	Tetanus^a^		Polio type 1^b^		Polio type 2^b^	Polio type 3^b^
		Girls	Boys	Girls	Boys		
Intercept	−1.07** (−1.45 to 0.68)	1.09** (0.55–1.64)	1.30** (0.73–1.86)	9.20** (8.17–10.24)	8.87** (8.04–9.71)	9.72** (9.04–10.40)	9.41** (8.63–10.18)
Winter vaccination	ref.	ref.	ref.	ref.	ref.	ref.	ref.
Spring vaccination	−0.23 (−0.63 to 0.16)	−0.28 (−0.85 to 0.29)	−0.14 (−0.89 to 0.61)	0.004 (−1.08 to 1.09)	0.09 (−0.81 to 0.98)	−0.22 (−0.91 to 0.48)	−0.23 (−1.03 to 0.57)
Summer vaccination	−0.45* (−0.85 to 0.05)	−0.33 (−0.88 to 0.22)	−1.28* (−2.27 to 0.28)	−0.50 (−1.55 to 0.56)	−0.24 (−1.18 to 0.70)	−0.72* (−1.42 to 0.01)	−0.83* (−1.64 to 0.02)
Autumn vaccination	−0.23 (−0.64 to 0.18)	−0.24 (−0.81 to 0.33)	−0.02 (−0.96 to 0.92)	0.06 (−1.02 to 1.15)	−0.81 (−1.75 to 0.12)	−0.53 (−1.25 to 0.18)	−0.48 (−1.30 to 0.34)
[1] Time post-vaccination^c^	−0.05** (−0.07 to 0.04)	−0.06** (−0.08 to 0.05)	−0.09** (−0.11 to 0.06)	−0.11** (−0.14 to 0.08)	−0.12** (−0.15 to 0.09)	−0.15** (−0.17 to 0.12)	−0.20** (−0.22 to 0.17)
Girl	0.07 (−0.21 to 0.36)	–	–	–	–	0.72** (0.23–1.22)	0.54 (−0.03 to 1.11)
Winter vaccination × [1]	–	–	ref.	–	–	–	–
Spring vaccination × [1]	–	–	0.01 (−0.03 to 0.04)	–	–	–	–
Summer vaccination × [1]	–	–	0.06** (0.02 to 0.11)	–	–	–	–
Autumn vaccination × [1]	–	–	0.01 (−0.03 to 0.06)	–	–	–	–
Observations	434	197	237	197	237	434	434
Adjusted *R*^2^	0.14	0.21	0.28	0.20	0.22	0.29	0.35
Residual standard error	1.48 (df = 428)	1.34 (df = 192)	1.26 (df = 229)	2.56 (df = 192)	2.56 (df = 232)	2.60 (df = 428)	2.98 (df = 428)
*F* statistic	15.37** (df = 5; 428)	14.01** (df = 4; 192)	14.19** (df = 7; 229)	13.07** (df = 4; 192)	17.60** (df = 4; 232)	36.65** (df = 5; 428)	48.28** (df = 5; 428)

*Notes*: Confidence intervals (95% CI) in parentheses; reference levels are winter (season of vaccination) and boy (sex).

a Log-transformed.

b Log 2-transformed.

c In months.

Levels of significance: **P < 0.01; *P < 0.05.

**Table 4.** Linear multivariate regression model estimates for antibody levels as a function of season of vaccination, time post-vaccination and sex for MMR sub-sample, Pienter-2 study

	Measles^a^	Mumps^a^	Rubella^a^
Intercept	1.10** (0.88–1.32)	4.86** (4.61–5.10)	4.59** (4.24–4.95)
Winter vaccination	ref.	ref.	ref.
Spring vaccination	−0.07 (−0.30 to 0.16)	−0.04 (−0.29 to 0.21)	0.18 (−0.27 to 0.64)
Summer vaccination	−0.09 (−0.32 to 0.14)	−0.02 (−0.27 to 0.24)	0.22 (−0.25 to 0.68)
Autumn vaccination	−0.15 (−0.38 to 0.07)	−0.27* (−0.51 to 0.02)	−0.48* (−0.92 to 0.04)
[1] Time post-vaccination^b^	−0.01** (−0.01 to 0.01)	−0.003* (−0.01 to 0.0001)	−0.01** (−0.02 to 0.002)
Girl	0.11 (−0.04 to 0.26)	0.23** (0.07–0.40)	0.32** (0.16–0.48)
Winter vaccination × [1]	–	–	ref.
Spring vaccination × [1]	–	–	−0.01* (−0.02 to 0.001)
Summer vaccination × [1]	–	–	−0.01* (−0.02 to 0.002)
Autumn vaccination × [1]	–	–	−0.0005 (−0.01 to 0.01)
Observations	811	811	811
Adjusted *R*^2^	0.07	0.02	0.14
Residual standard error	1.11 (df = 805)	1.21 (df = 805)	1.13 (df = 802)
*F* statistic	14.10** (df = 5; 805)	3.54** (df = 5; 805)	17.97** (df = 8; 802)

*Notes*: Confidence intervals (95% CI) in parentheses; reference levels are winter (season of vaccination) and boy (sex).

a Log-transformed.

b In months.

Levels of significance: **P < 0.01; *P < 0.05.

**Table 5.** Linear multivariate regression model estimates for antibody levels as a function of season of vaccination, time post-vaccination and sex for MenC sub-sample, Pienter-2 study

	MenC^a^
Intercept	0.69** (0.27–1.10)
Winter vaccination	ref.
Spring vaccination	0.41 (−0.004 to 0.83)
Summer vaccination	0.16 (−0.28 to 0.60)
Autumn vaccination	0.45* (0.05–0.85)
Time post-vaccination^b^	−0.07** (−0.09 to 0.06)
Girl	0.20 (−0.08 to 0.48)
Observations	311
Adjusted *R*^2^	0.26
Residual standard error	1.24 (df = 305)
*F* statistic	22.33** (df = 5; 305)

*Notes*: Confidence intervals (95% CI) in parentheses; reference levels are winter (season of vaccination) and boy (sex).

a Log-transformed.

b In months.

Levels of significance: **P < 0.01; *P < 0.05.

When considering a less stringent significance level (at *α* = 0.05 level), we observed that children vaccinated in summer had lower antibody levels against diphtheria (36% lower; *β* = −0.45; *P* = 0.029), against poliovirus serotype 2 (51% lower; *β* = −0.72; *P* = 0.048), and against poliovirus serotype 3 (56% lower; *β* = −0.83; *P* = 0.045) compared to children vaccinated in winter. For boys, summer vaccination induced tetanus antibody levels directly post-vaccination 72% lower (*β* = −1.28; *P* = 0.013) in comparison with winter-vaccinated boys (Table 3). Children vaccinated in autumn had rubella antibody levels directly post-vaccination 38% lower (*β* = −0.48; *P* = 0.031) compared to winter-vaccinated children. Autumn vaccination induced mumps antibody levels over the 3-year ‘follow-up’ period 24% lower (*β* = −0.27; *P* = 0.032) than winter vaccination. The rate of rubella antibodies decline over time was 1% per month faster when children were vaccinated in summer (*β* = −0.01; *P* = 0.021) or spring (*β* = −0.01; *P* = 0.032), compared to winter vaccination (Table 4). Autumn vaccination induced MenC antibody levels over the 3-year ‘follow-up’ period 57% higher (*β* = 0.45; *P* = 0.030), when compared to winter vaccination (Table 5).

### Sex differences

The antibody response induced against polio type 2 was overall higher in girls than in boys (Table 3: *β* = 0.72; *P* < 0.01; Supplementary Fig. S1D). Furthermore, overall antibody levels against mumps and rubella were higher in girls than in boys (Table 4: *β* = 0.23 and *β* = 0.32, respectively; *P* < 0.01), but not for measles. This is also visualised in Supplementary Figure S1F–H.

### Correlation of antibody levels against different pathogens within individuals

The antibody levels elicited by the different components of combination vaccines correlated moderately within children: the pairwise Pearson correlation of antibody levels induced by MMR and DT-IPV combination vaccines was ⩾0.39 and ⩾0.41, respectively (*P* ⩽ 0.001). However, the antibody levels elicited by different vaccines administered on the same day (i.e. MMR and MenC) showed a different pattern: MenC levels did not correlate with measles (*r* = 0.12, *P* = 0.03), mumps (*r* = 0.08, *P* = 0.15) or rubella antibody levels (*r* = 0.14, *P* = 0.01) (Supplementary Table S2).

### Sensitivity analyses

The sensitivity analysis excluding oversampled non-Western children (Supplementary Tables S3–S5) showed no seasonal association anymore. It yielded only one significant term at *α* = 0.01 level: rubella antibody levels were higher in girls than in boys (*β* = 0.27, *P* < 0.01; Supplementary Table S4).

A model accounting for a possible biphasic decay of antibodies post-vaccination only improved the model fit to the diphtheria antibodies (adj. *R*^2^ from 0.14 to 0.21), and to tetanus antibodies in boys (adj. *R*^2^ from 0.28 to 0.31), compared with the best-fit models assuming a monophasic antibody decay (Supplementary Table S6). These models did not show a significant association between antibody levels and season of vaccination against diphtheria nor for tetanus (data not shown).

## Discussion

We found little evidence to support the hypothesis that season of vaccination in children may be associated with different antibody levels for the diseases investigated in a nationwide cohort from the Netherlands. The only indication for a seasonal association at the conservative significance threshold of 0.01 was found for boys vaccinated against tetanus, who showed a slower decline in antibody levels after vaccination in summer compared to vaccination in winter. However, the association between season of vaccination and tetanus antibody levels seems to be better modelled when accounting for a biphasic decay of antibodies post-vaccination, where the association is then no longer present. At a less conservative significance level (*P*-value <0.05), we found some tendency of association between season of vaccination and antibody levels for all the diseases investigated – except for poliovirus serotype 1 and measles – with associations for mumps, rubella and diphtheria having a *P*-value around 0.03.

To our knowledge, our study is the first to investigate the impact of season of vaccination on antibodies against mumps and MenC. Moreover, no studies have focused on interaction effects involving season of vaccination, sex and time post-vaccination.

A study by Moore *et al*. did not find an association between season of vaccination and tetanus and diphtheria antibody levels in a cohort of 138 infants in The Gambia [42]. Antibody levels were measured and compared immediately before the two first doses of tetanus and diphtheria vaccines were administered to children at 8 and 16 weeks of age. We investigated immunity in older children, over a broader age-range, hampering comparisons.

Consistent with a report from the Netherlands [14], season of vaccination did not influence measles antibody levels in our study. In a study in Guinea-Bissau, however, in a cohort of 415 children, those vaccinated during the rainy season at 9 months of age had higher measles antibody levels at 24 months of age than those vaccinated in the dry season [43]. In a study with 203 children aged 4–5 years, Linder *et al*. found a stronger immune response to rubella vaccine at 12 months in children vaccinated in winter in Israel [44]. On the other hand, the previous report in the Netherlands could not establish differences in rubella antibody levels related to season of vaccination at 14 months, in a cohort of 719 children aged 2–7 years old [14], just as in our study.

### Correlation of antibody levels against different pathogens within individuals

Although MMR and MenC vaccines are administered to children on the same day, the individual's antibody levels did not show similar immunogenicity patterns by season of vaccination. Differences in the magnitude of responses induced by different vaccines administered on the same day (i.e. same season) confirm the idea that the response – and potentially the effect of seasonality – is antigen-specific and is not linked to an individual's capacity to mount/produce an immune response. When present, the impact of seasonality on the vaccine-induced response seems to be pathogen-specific, i.e. the magnitude of the response elicited by some vaccines seems to be more affected by the seasonal effect than others. Different pathogens engage the immune response through different pathways, which may themselves be differentially affected by season.

### Sensitivity analyses

The seasonal effect found for boys vaccinated against tetanus was no longer statistically significant when we performed sensitivity analysis excluding the oversampled children: this could result from a loss of power due to smaller sample size, or it might indicate that non-Western children are subject to a stronger seasonal effect, for instance due to re-exposure to pathogens when travelling to their homeland, different behaviour regarding sun light exposure, or genetic differences impacting their immune function [45, 46]. This is in line with the higher GMT/C found in oversampled children for all antibody levels (except for measles), when compared to the levels of the national sample (data not shown), and as previously reported in the non-Western community in the Netherlands [24].

We assumed a linear decay of log(antibody levels), which could lead to over- or underestimation of model estimates if in fact vaccination would induce different antibody populations with varying decay rates [47]. The latter might entail a biphasic decay of log(antibody levels). Therefore, we compared the model fit of the linear models with an alternative model including log-transformed ‘time post-vaccination’, which showed that only the model fit of diphtheria and tetanus (boys) benefited from the inclusion of the non-linear independent variable. It is interesting to note that the linear model for which we found the strongest association between antibody levels and season of vaccination (tetanus antibody decline over time in boys) might be better modelled with a biphasic decay, and therefore may no longer present that association. Nevertheless, it is fair to assume a linear antibody decay for the vaccines that did not have their model fit improved by the inclusion of a biphasic decay of log(antibody levels). Thus, linear models that showed tendencies of association between season of vaccination and immunity (i.e. *P*-value <0.05) could still be used and might produce increased significance levels with a larger sample size.

### Variables not controlled for

We did not control for maternal antibody status, since children older than 9 months (the minimum age included in our sub-samples) are likely no longer protected by maternal antibodies [48]. Moreover, despite the fact that infant age may largely influence the immune response to a vaccine [4], age at vaccination had small variance and low predictive power in our dataset, and its inclusion in the models could cause precision issues and poor performance. This limited variability in timing of vaccination means we cannot disentangle the association between season of vaccination from the association with season of birth.

Even though some literature suggests that vaccine administration timing (e.g. morning or afternoon) might impact the induced immune response [7], we do not believe that this effect played a role in our study, since vaccines were routinely randomly administered throughout the day, and therefore, vaccine administration timing is not likely to vary across seasons.

Our results were not corrected for socioeconomic status, which could be a proxy for factors that might influence antibody levels, such as nutritional status. Nonetheless, nutritional status is not likely to vary by season of vaccination in children in the Netherlands, as opposed to countries where the supply of food is reduced in some periods of the year.

### Limitations

A limitation of our analysis is the cross-sectional nature of the study, which does not control for the influence of individual heterogeneity in antibody waning rates. We compared the decay rates obtained in the current study at the population level with estimated waning rates of MMR antibody levels in a longitudinal cohort study of 43 children from the Netherlands over 3 years [49]. The decline in rubella antibody levels was the only waning rate comparable to our study (Supplementary Table S7; *β*_time post-vaccination_ = −0.01 ln IU/ml per month after vaccination, for both studies). The waning rate for measles antibody levels was not statistically significant and mumps antibody levels showed an increase over time (Supplementary Table S7; *β*_time post-vaccination_ = 0.02 ln IU/ml per month after vaccination) in the longitudinal study. It is noteworthy that a booster dose of DT(a)P-IPV vaccine is recommended at 4 years old, which is around the latest longitudinal sampling moment in the above cohort study and could have non-specifically enhanced measles and mumps antibody levels, as this was also observed for other vaccines [50]. We believe that the same effect would not impact our main findings, as a large range of age groups was sampled. In addition, participants of the longitudinal study might have been heterogeneously exposed to measles virus, since most of them were born during or shortly before a measles outbreak in the Netherlands in 2013 [51], or ultimately, the sample size did not provide sufficient statistical power to estimate the waning rates. Nevertheless, considering that our cross-sectional observations followed a longitudinal pattern and that studies observed comparable trends in immune response per time after exposure in cross-sectional and longitudinal data (individual basis) [52], we believe that our cross-sectional design provides reasonable estimates of waning antibody rates.

We assumed the environmental exposure to pathogens was minimal, based on national notification systems and surveillance information. Moreover, we excluded children from areas where certain pathogens were more likely to circulate. It is noteworthy that vaccine responses against pathogens not circulating extensively in the environment (e.g. diphtheria and poliomyelitis in the Netherlands) may be suitable biomarkers for analysis of seasonality of immunity, since the seasonal effect of the vaccine on antibody levels is unlikely to be influenced by the effect of seasonal circulation of the pathogen.

We also assumed that differences in the ambient temperature did not affect the vaccines' cold chain, as it could have been the case in countries with higher average yearly temperatures.

Although for tetanus, diphtheria, poliomyelitis, measles and rubella, antibody levels are good correlates of protection, and MenC IgG antibody levels correlate with the ‘gold standard’ (serum bactericidal assay), mumps antibodies poorly correlate with immunological protection [53]. Therefore, differences in mumps antibody levels, whether related to season of vaccination or not, may not result in altered immunological protection.

It is known that the length of time between vaccine doses impacts the antibody production following the latest one [54], and thus when multiple doses are given within a brief period, teasing out the contribution of each dose in shaping the magnitude of the immune response is practically unattainable. Therefore, although we selected the vaccination moment at which the largest variation between pre- and post-vaccination antibody levels occurs, we were unable to disentangle the impact of the season of the previous DT-IPV doses on children's antibody levels.

In conclusion, our findings provide limited evidence that season of vaccination in children may be associated with antibody levels against the different pathogens investigated in a nationwide cohort from the Netherlands. Moreover, antibody levels do not seem to fall below protection levels over the study period considered, so clinical implications might be limited. We have applied a conservative significance threshold of 0.01; however, it is noteworthy that associations between season of vaccination and immunity against mumps, rubella and diphtheria had a *P*-value around 0.03. Possibly, adding the data obtained in the last nationwide serological study in the Netherlands (Pienter-3) could provide the power required to increase significance level of the results. Thus, linear models that showed tendencies of association between season of vaccination and immunity (i.e. *P*-value <0.05) could still be used and might produce increased significance levels with a larger sample size. In addition, further longitudinal immunological and epidemiological investigation of seasonality of vaccine-induced immunity in countries of temperate climate is needed to confirm our findings, to assess clinical relevance and to help understand the possible underlying mechanisms involved.

## Data Availability

The data that support the findings of this study are available on request from the authors.

## Supplementary material

For supplementary material accompanying this paper visit https://doi.org/10.1017/S0950268820002691. click here to view supplementary material
