# Supplementary material for: Association between season of vaccination and antibody levels against infectious diseases
Source: Epidemiol Infect. 2020 Nov 5;148:e276. doi: 10.1017/S0950268820002691 (PMC7770373; doi:10.1017/S0950268820002691)
Supplement: Supplementary file 1 [file S0950268820002691sup.zip › S0950268820002691sup001.docx]

**SUPPLEMENTARY MATERIAL**

**Supplementary Figure 1. Observed antibody levels against time post-vaccination, by season of vaccination per pathogen. Regression lines predicted by the final models per sex per pathogen, Pienter-2 Study.**

**
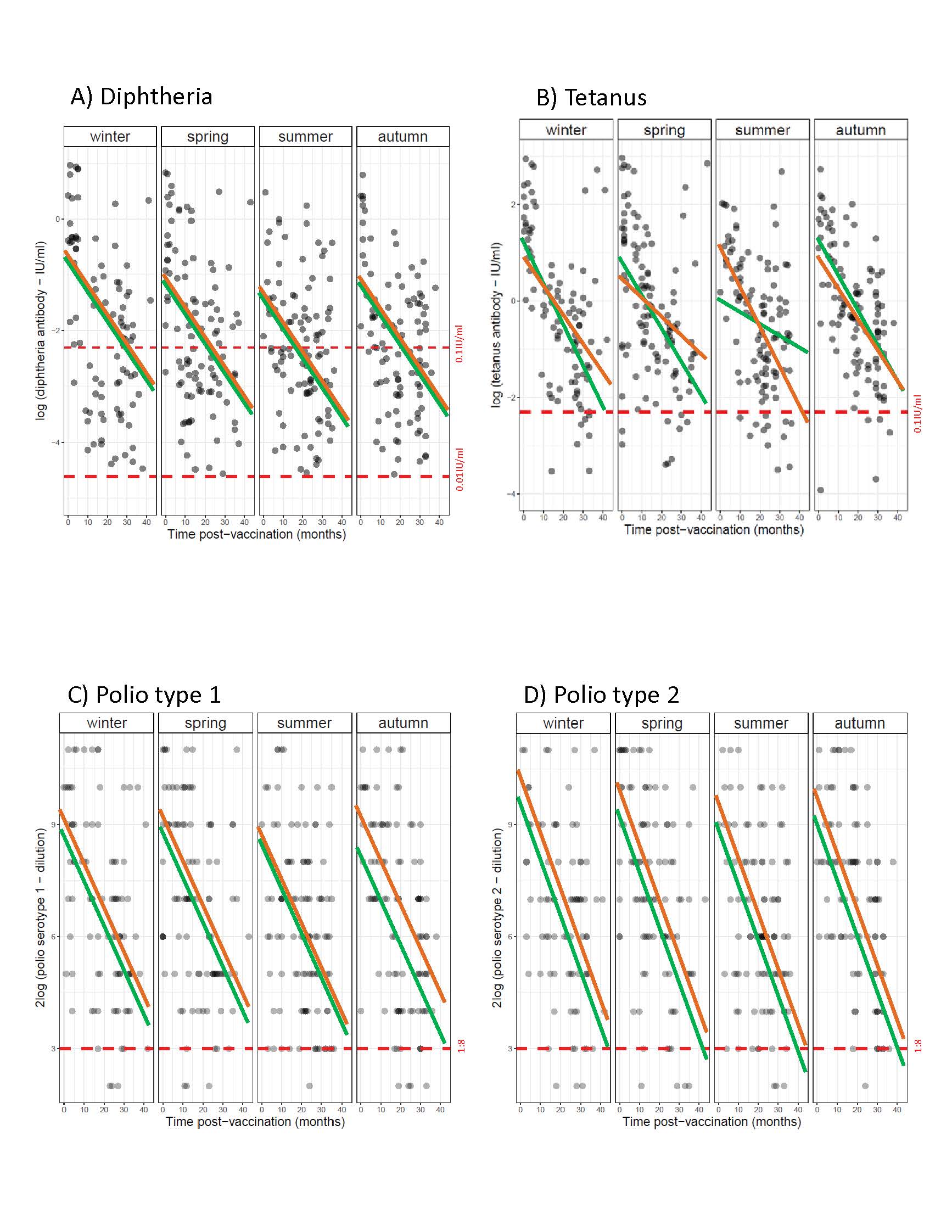
**

Sex : girl boy

Note: Darker points correspond to overlap of observations; Dashed horizontal red lines are thresholds of protection.

.

**
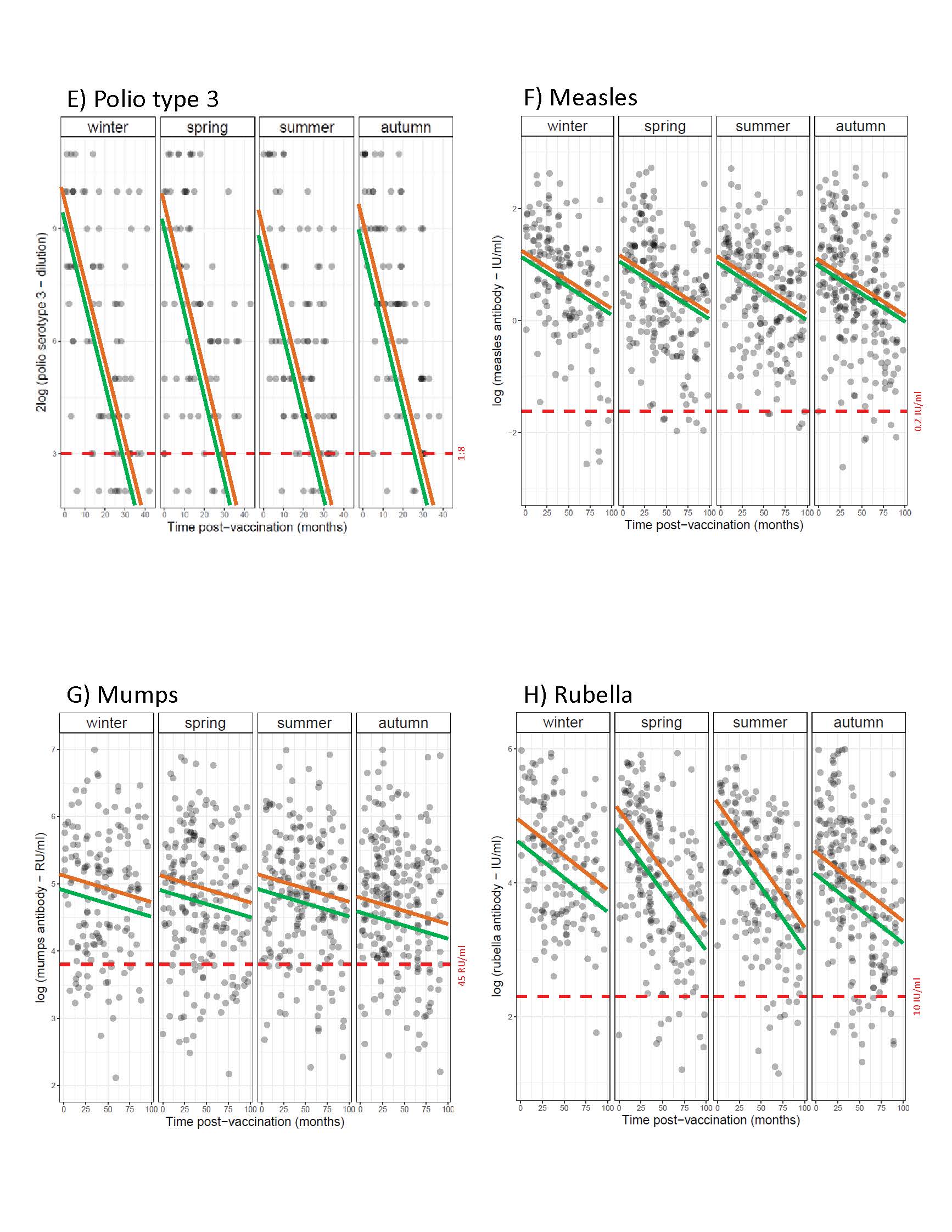
**

Sex: girl boy

Note: Darker points correspond to overlap of observations; Dashed horizontal red lines are thresholds of protection.

**
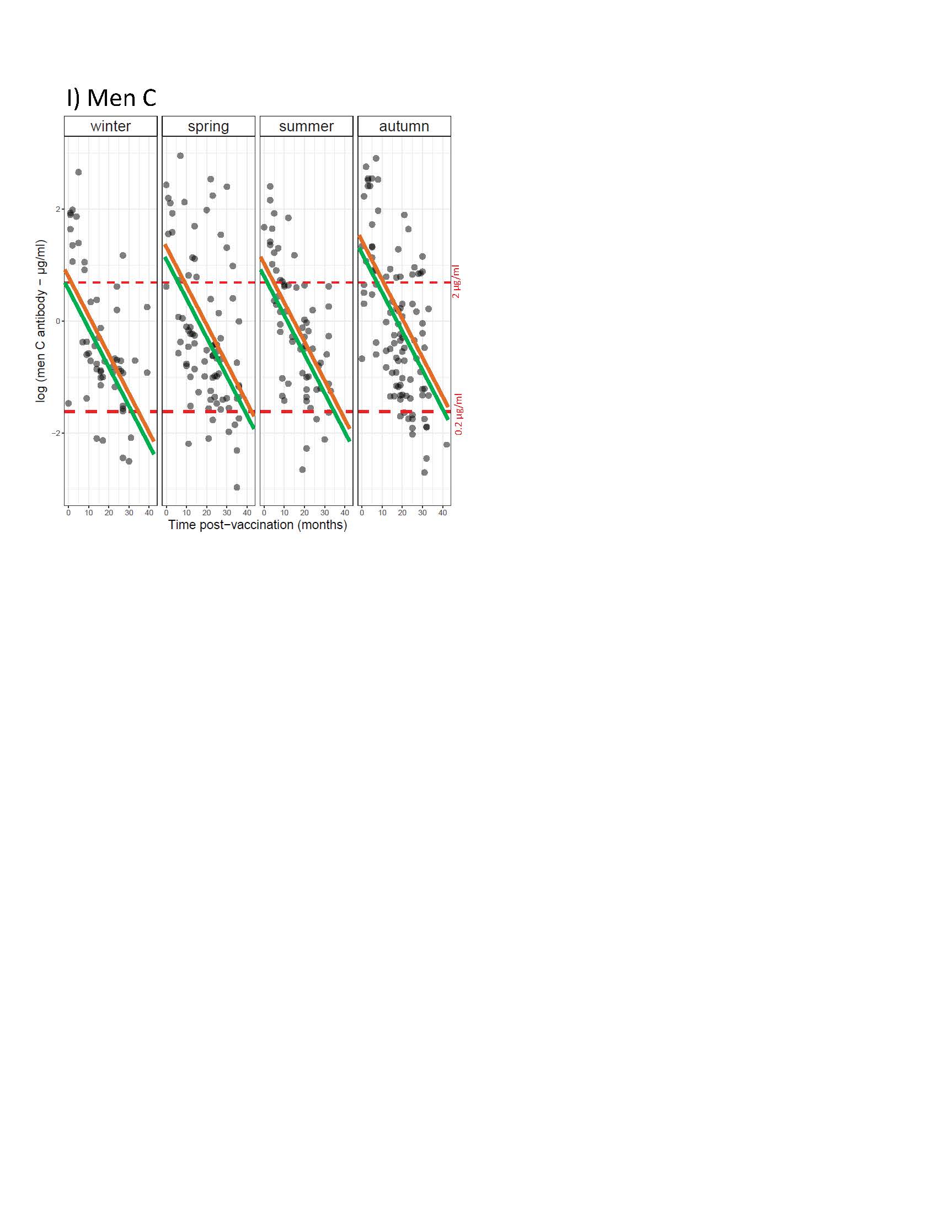
**

Sex: girl boy

Note: Darker points correspond to overlap of observations; Dashed horizontal red lines are thresholds of protection.

| \| **Supplementary Table 1. Seroconversion rate per season of vaccination, Pienter-2 Study.** \| \| \| \| \| \| \| \| \| \| \| \| \| \| \| \| \| --- \| --- \| --- \| --- \| --- \| --- \| --- \| --- \| --- \| --- \| --- \| --- \| --- \| --- \| --- \| --- \| \|  \| \| ***Seroconversion threshold*** \| \| ***Winter*** \| \| ***Spring*** \| \| ***Summer*** \| \| ***Autumn*** \| \| ***Overall*** \| \| ***p-value^a^*** \| \| Diphtheria \| 0.01 IU/ml \| \| 93% \| \| 91% \| \| 92% \| \| 97% \| \| 93% \| \| 0.249 \| \| \| \| \| Tetanus \| 0.1 IU/ml \| \| 93% \| \| 91% \| \| 92% \| \| 97% \| \| 93% \| \| 0.424 \| \| \| \| \| Polio type 1 \| 1:8 \| \| 92% \| \| 93% \| \| 97% \| \| 91% \| \| 93% \| \| 0.291 \| \| \| \| \| Polio type 2 \| 1:8 \| \| 93% \| \| 91% \| \| 92% \| \| 91% \| \| 92% \| \| 0.936 \| \| \| \| \| Polio type 3 \| 1:8 \| \| 77% \| \| 75% \| \| 71% \| \| 71% \| \| 73% \| \| 0.677 \| \| \| \| \| Measles \| 0.2 IU/ml \| \| 95% \| \| 97% \| \| 97% \| \| 96% \| \| 96% \| \| 0.932 \| \| \| \| \| Mumps \| 45 RU/ml \| \| 85% \| \| 81% \| \| 85% \| \| 80% \| \| 82% \| \| 0.372 \| \| \| \| \| Rubella \| 10 IU/ml \| \| 99% \| \| 94% \| \| 94% \| \| 90% \| \| 93% \| \| 0.010 \| \| \| \| \| MenC \| 0.2 µg/ml \| \| 88% \| \| 88% \| \| 90% \| \| 87% \| \| 88% \| \| 0.594 \| \| \| \|   ***^a^****Chi-square test* |  |  |  |
| --- | --- | --- | --- | --- | --- | --- | --- | --- | --- | --- | --- | --- | --- | --- | --- | --- | --- | --- | --- | --- | --- | --- | --- | --- | --- | --- | --- | --- | --- | --- | --- | --- | --- | --- | --- | --- | --- | --- | --- | --- | --- | --- | --- | --- | --- | --- | --- | --- | --- | --- | --- | --- | --- | --- | --- | --- | --- | --- | --- | --- | --- | --- | --- | --- | --- | --- | --- | --- | --- | --- | --- | --- | --- | --- | --- | --- | --- | --- | --- | --- | --- | --- | --- | --- | --- | --- | --- | --- | --- | --- | --- | --- | --- | --- | --- | --- | --- | --- | --- | --- | --- | --- | --- | --- | --- | --- | --- | --- | --- | --- | --- | --- | --- | --- | --- | --- | --- | --- | --- | --- | --- | --- | --- | --- | --- | --- | --- | --- | --- | --- | --- | --- | --- | --- | --- | --- | --- | --- | --- | --- | --- | --- | --- | --- | --- | --- | --- | --- | --- | --- | --- | --- | --- | --- | --- | --- | --- | --- | --- | --- | --- | --- | --- | --- | --- | --- | --- | --- | --- | --- | --- | --- | --- | --- | --- | --- | --- | --- | --- | --- | --- | --- | --- | --- | --- | --- | --- |

| **Supplementary Table 2. Pairwise Pearson correlation between individuals’ antibody levels, Pienter-2 Study.** | | | | | | | | | | | | | | |
| --- | --- | --- | --- | --- | --- | --- | --- | --- | --- | --- | --- | --- | --- | --- |
| ***Antibody*** | *1* | *2* | *3* | *4* | | *5* | | *6* | | *7* | | *8* | | *9* |
| *1 Diphtheria* | 1.00*** |  |  |  | |  | |  | |  | |  | |  |
| *2 Tetanus* | 0.69*** | 1.00*** |  |  | |  | |  | |  | |  | |  |
| *3 Polio type 1* | 0.46*** | 0.50*** | 1.00*** |  | |  | |  | |  | |  | |  |
| *4 Polio type 2* | 0.51*** | 0.57*** | 0.63*** | 1.00*** | |  | |  | |  | |  | |  |
| *5 Polio type 3* | 0.41*** | 0.53*** | 0.62*** | 0.66*** | | 1.00*** | |  | |  | |  | |  |
| *6 Measles* | 0.06 | 0.01 | 0.03 | -0.01 | | -0.08 | | 1.00*** | |  | |  | |  |
| *7 Mumps* | 0.05 | -0.06 | -0.05 | -0.03 | | -0.04 | | 0.39*** | | 1.00*** | |  | |  |
| *8 Rubella* | 0.07 | -0.03 | -0.06 | 0.001 | | -0.01 | | 0.50*** | | 0.52*** | | 1.00*** | |  |
| *9 MenC* | 0.21*** | 0.28*** | 0.15** | 0.21*** | | 0.25*** | | 0.12* | | 0.08 | | 0.14* | | 1.00*** |
|  | | | | |  | |  | |  | |  | |  | |
| Levels of significance: ***p< 0.001; **p< 0.01; *p< 0.05  Level of significance adjusted for multiple testing (Bonferroni correction): ***p< 0.001 (0.05/45 tests) | | | | | | | | | | | | | | |

| **Supplementary Table 3. Sensitivity analysis excluding oversampled migrants: Linear Multivariate Regression Model estimates for antibody levels as a function of season of vaccination, time post-vaccination and sex for DT-IPV sub-sample, Pienter-2 Study.** | | | | | | | | | | | | | | | | | | |
| --- | --- | --- | --- | --- | --- | --- | --- | --- | --- | --- | --- | --- | --- | --- | --- | --- | --- | --- |
|  | ***Diphtheria^a^*** | | | ***Tetanus^a^*** | | | | | ***Polio type 1^b^*** | | | | | | ***Polio type 2^b^*** | | | ***Polio type 3^b^*** |
|  |  |  |  | *girls* | | | *boys* | | | *girls* | | *boys* | | |  |  |  |  |
| *Intercept* | -1.02** (-1.47, -0.58) | | 0.90** (0.33, 1.47) | | | 1.49** (0.91, 2.07) | | 8.63** (7.37, 9.90) | | | 8.92** (7.94, 9.89) | | | 9.69** (8.90, 10.47) | | | 9.38** (8.49, 10.27) | |
| *Winter vaccination* | *ref.* | | *ref.* | | | *ref.* | | *ref.* | | | *ref.* | | | *ref.* | | | *ref.* | |
| *Spring vaccination* | -0.44 (-0.91, 0.02) | | -0.22 (-0.81, 0.37) | | | -0.01 (-0.85, 0.84) | | 0.40 (-0.89, 1.69) | | | -0.20 (-1.31, 0.92) | | | -0.44 (-1.27, 0.39) | | | -0.53 (-1.47, 0.41) | |
| *Summer vaccination* | -0.52* (-0.99, -0.06) | | 0.10 (-0.48, 0.68) | | | -1.29* (-2.40, -0.18) | | -0.05 (-1.32, 1.23) | | | -0.71 (-1.84, 0.42) | | | -0.72* (-1.55, 0.11) | | | -0.98* (-1.92, -0.05) | |
| *Autumn vaccination* | -0.25 (-0.73, 0.22) | | 0.28 (-0.34, 0.90) | | | -0.17 (-1.20, 0.85) | | 0.23 (-1.15, 1.60) | | | -0.60 (-1.70, 0.49) | | | -0.13 (-0.97, 0.71) | | | -0.45 (-1.40, 0.51) | |
| *[1] Time post-vaccination****^c^*** | -0.06** (-0.08, -0.04) | -0.08** (-0.09, -0.06) | | | -0.09** (-0.12, -0.06) | | | -0.11** (-0.15, -0.07) | | | -0.12** (-0.16, -0.08) | | | -0.16** (-0.19, -0.14) | | | -0.21** (-0.24, -0.18) | |
| *Girl* | 0.01 (-0.33, 0.35) | | - | | | - | | - | | | - | | | 0.70* (0.11, 1.30) | | | 0.46 (-0.21, 1.14) | |
| *Winter vaccination x [1]* | *-* | | *-* | | | *ref.* | | *-* | | | *-* | | | *-* | | | *-* | |
| *Spring vaccination x [1]* | - | | - | | | -0.02 (-0.06, 0.03) | | - | | | - | | | - | | | - | |
| *Summer vaccination x [1]* | - | | - | | | 0.05 (-0.01, 0.10) | | - | | | - | | | - | | | - | |
| *Autumn vaccination x [1]* | - | | - | | | 0.01 (-0.03, 0.06) | | - | | | - | | | - | | | - | |
| Observations | 294 | | 134 | | | 160 | | 134 | | | 160 | | | 294 | | | 294 | |
| Adjusted R square | 0.17 | | 0.33 | | | 0.36 | | 0.17 | | | 0.2 | | | 0.33 | | | 0.38 | |
| Residual Standard Error | 1.45 (df = 288) | | 1.18 (df = 129) | | | 1.18 (df = 152) | | 2.61 (df = 129) | | | 2.59 (df = 155) | | | 2.57 (df = 288) | | | 2.91 (df = 288) | |
| F Statistic | 12.80** (df = 5; 288) | 17.34** (df = 4; 129) | | | 13.76** (df = 7; 152) | | | 7.58** (df = 4; 129) | | | 11.08** (df = 4; 155) | | | 29.27** (df = 5; 288) | | | 37.41** (df = 5; 288) | |
| ^a^log-transformed; ^b^log2-transformed; ^c^in months. Notes: Confidence Intervals (95% CI) in parentheses; Reference levels are winter (season of vaccination) and boy (sex); Levels of significance: **p< 0.01; *p< 0.05. | | | | | | | | | | | | | | | | | | |
|  | | | | | | | | | | | | |  | | |  | | |

| **Supplementary Table 4. Sensitivity analysis excluding oversampled migrants: Linear Multivariate Regression Model estimates for antibody levels as a function of season of vaccination, time post-vaccination and sex for MMR sub-sample, Pienter-2 Study.** | | | | | | |
| --- | --- | --- | --- | --- | --- | --- |
|  |  |  |  |  |  |  |
|  | | ***Measles^a^*** | | ***Mumps^a^*** | ***Rubella^a^*** | |
| *Intercept* | | 1.17** (0.93, 1.42) | | 4.82** (4.55, 5.08) | 4.48** (4.09, 4.87) | |
| *Winter vaccination* | | *ref.* | | *ref.* | *ref.* | |
| *Spring vaccination* | | -0.16 (-0.41, 0.09) | | -0.05 (-0.32, 0.23) | 0.22 (-0.28, 0.72) | |
| *Summer vaccination* | | -0.17 (-0.43, 0.09) | | 0.01 (-0.27, 0.29) | 0.21 (-0.30, 0.72) | |
| *Autumn vaccination* | | -0.24* (-0.48, 0.01) | | -0.22 (-0.48, 0.05) | -0.40 (-0.88, 0.08) | |
| *[1] Time post-vaccination****^b^*** | | -0.01** (-0.01, -0.01) | | -0.003 (-0.01, 0.0003) | -0.01* (-0.02, -0.001) | |
| *Girl* | | 0.13 (-0.03, 0.30) | | 0.23* (0.05, 0.42) | 0.27** (0.09, 0.44) | |
| *Winter vaccination x [1]* | | *-* | | *-* | *ref.* | |
| *Spring vaccination x [1]* | | - | | - | -0.01 (-0.02, 0.0002) | |
| *Summer vaccination x [1]* | | - | | - | -0.01 (-0.02, 0.001) | |
| *Autumn vaccination x [1]* | | - | | - | -0.0002 (-0.01, 0.01) | |
| Observations | | 617 | | 617 | 617 | |
| Adjusted R square | | 0.07 | | 0.01 | 0.13 | |
| Residual Standard Error | | 1.06 (df = 611) | | 1.15 (df = 611) | 1.10 (df = 608) | |
| F Statistic | | 9.81** (df = 5; 611) | | 2.69* (df = 5; 611) | 12.33** (df = 8; 608) | |
| ^a^log-transformed; ^b^in months. Notes: Confidence Intervals (95% CI) in parentheses; Reference levels are winter (season of vaccination) and boy (sex); Levels of significance: **p< 0.01; *p< 0.05 | | | | | | |
| **Supplementary Table 5. Sensitivity analysis excluding oversampled migrants: Linear Multivariate Regression Model estimates for antibody levels as a function of season of vaccination, time post-vaccination and sex for MenC sub-sample, Pienter-2 Study.** | | | | |  |  |
|  | | ***MenC^a^*** | | |  |  |
| *Intercept* | | 0.57* (0.13, 1.02) | | |  |  |
| *Winter vaccination* | | *ref.* | | |  |  |
| *Spring vaccination* | | 0.42 (-0.03, 0.88) | | |  |  |
| *Summer vaccination* | | 0.15 (-0.32, 0.63) | | |  |  |
| *Autumn vaccination* | | 0.47* (0.03, 0.92) | | |  |  |
| *Time post-vaccination****^b^*** | | -0.07** (-0.09, -0.06) | | |  |  |
| *Girl* | | 0.19 (-0.12, 0.49) | | |  |  |
| Observations | | 266 | | |  |  |
| Adjusted R square | | 0.24 | | |  |  |
| Residual Standard Error | | 1.25 (df = 260) | | |  |  |
| F Statistic | | 17.86** (df = 5; 260) | | |  |  |
| ^a^log-transformed; ^b^in months. Notes: Confidence Intervals (95% CI) in parentheses; Reference levels are winter (season of vaccination) and boy (sex); Levels of significance: **p< 0.01; *p< 0.05 | | | | |  |  |
|  | | | | |  |  |

| **Supplementary Table 6. Comparative results of model fit (adjusted R square): Time post-vaccination (original scale) *versus* Time post-vaccination (log-transformed), Pienter-2 Study.** | | | |
| --- | --- | --- | --- |
|  |  | *Final model adjusted R square* | |
|  |  | **Time post-vaccination (original scale)** | **Time post-vaccination (log-transformed)** |
| Diphtheria^a^ |  | 0.14 | 0.21 |
| Tetanus^a^ | *girls* | 0.21 | 0.21 |
|  | *boys* | 0.28 | 0.31 |
| Polio type 1^b^ | *girls* | 0.20 | 0.20 |
|  | *boys* | 0.22 | 0.19 |
| Polio type 2^b^ |  | 0.29 | 0.27 |
| Polio type 3^b^ |  | 0.35 | 0.33 |
| Measles^a^ |  | 0.07 | 0.03 |
| Mumps^a^ |  | 0.02 | 0.01 |
| Rubella^a^ |  | 0.14 | 0.10 |
| MenC^a^ |  | 0.26 | 0.25 |
| ^a^log-transformed; blog2-transformed | | | |

| **Supplementary Table 7. Comparative results of estimates of waning MMR antibodies: Linear Mixed Model (longitudinal cohort) *versus* Linear Multivariate Regression Model, Pienter-2 Study** | | | | | | | | | |
| --- | --- | --- | --- | --- | --- | --- | --- | --- | --- |
|  |  | ***Measles^a,d^*** | |  | ***Mumps^a,d^*** | |  | ***Rubella^a,d^*** | |
|  |  | *Longitudinal cohort* | *Pienter-2 Study* |  | *Longitudinal cohort* | *Pienter-2 Study* |  | *Longitudinal cohort* | *Pienter-2 Study* |
| *Intercept* |  | 0.85** (0.34, 1.35) | 1.12* (0.91, 1.32) |  | 4.08** (3.69, 4.47) | 4.82* (4.59, 5.05) |  | 5.24* (4.91, 5.57) | 4.48* (4.27, 4.70) |
| *Time post-vaccination^b^* |  | 0.0001 (-0.009, 0.009) | -0.01* (-0.02, -0.01) |  | 0.02** (0.01, 0.03) | -0.003 (-0.01, 0.001) |  | -0.01** (-0.02, -0.01) | -0.01* (-0.02, -0.01) |
| *Girl* |  | 0.16 (-0.52, 0.84) | 0.11 (-0.07, 0.29) |  | 0.86** (0.35, 1.38) | 0.19 (-0.01, 0.39) |  | 0.18 (-0.34, 0.55) | 0.24* (0.05, 0.43) |
| Observations |  | 106 | 483^c^ |  | 103 | 483^c^ |  | 106 | 483^c^ |
| ^a^log-transformed; ^b^in months; ^c^Children aged between 15 to 50 months were included in the analysis for comparability purposes; ^d^Models adjusted for sex only. | | | | | | | | | |
| Notes: Confidence Intervals (95% CI) in parentheses; Reference levels are winter (season of vaccination) and boy (sex); Levels of significance: **p< 0.01; *p< 0.05 | | | | | | | | |  |
