# Supplementary figures and images for: Association between season of vaccination and antibody levels against infectious diseases
Source: Epidemiol Infect. 2020 Nov 5;148:e276. doi: 10.1017/S0950268820002691 (PMC7770373; doi:10.1017/S0950268820002691)

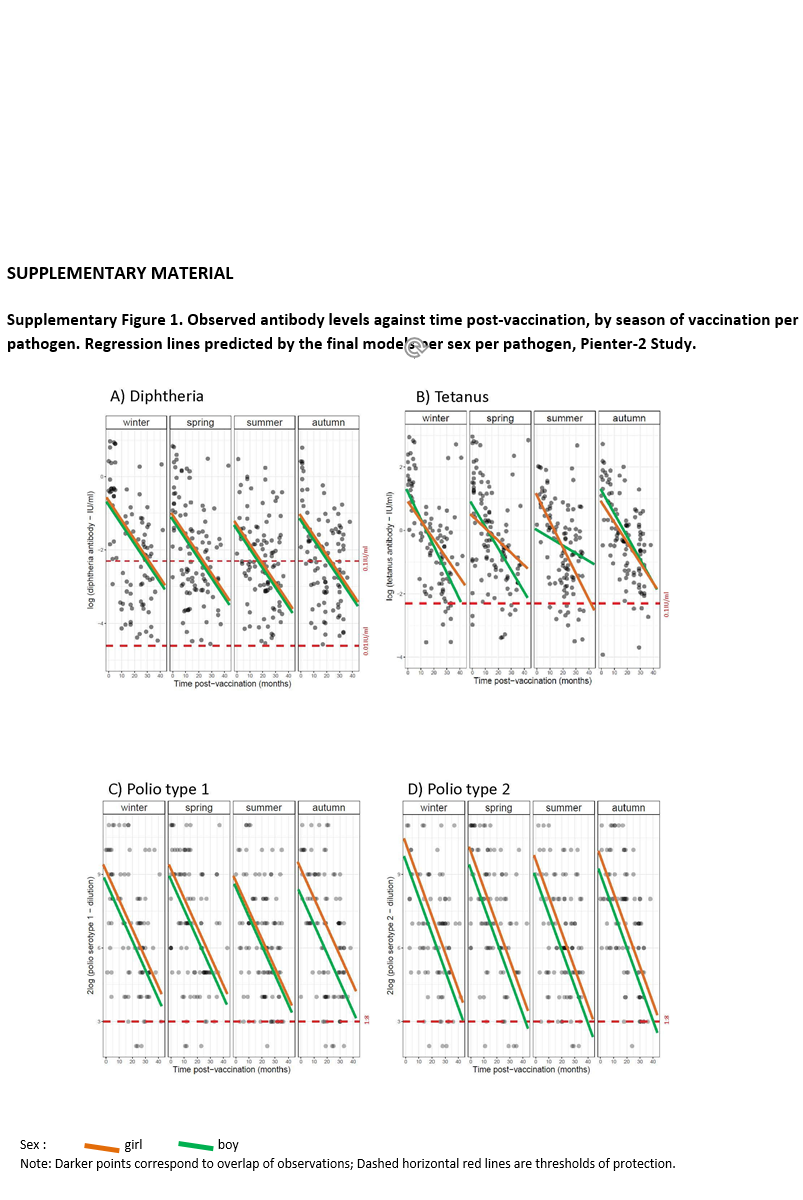

Supplement: Supplementary file 1 [file S0950268820002691sup.zip › S0950268820002691sup002.png]

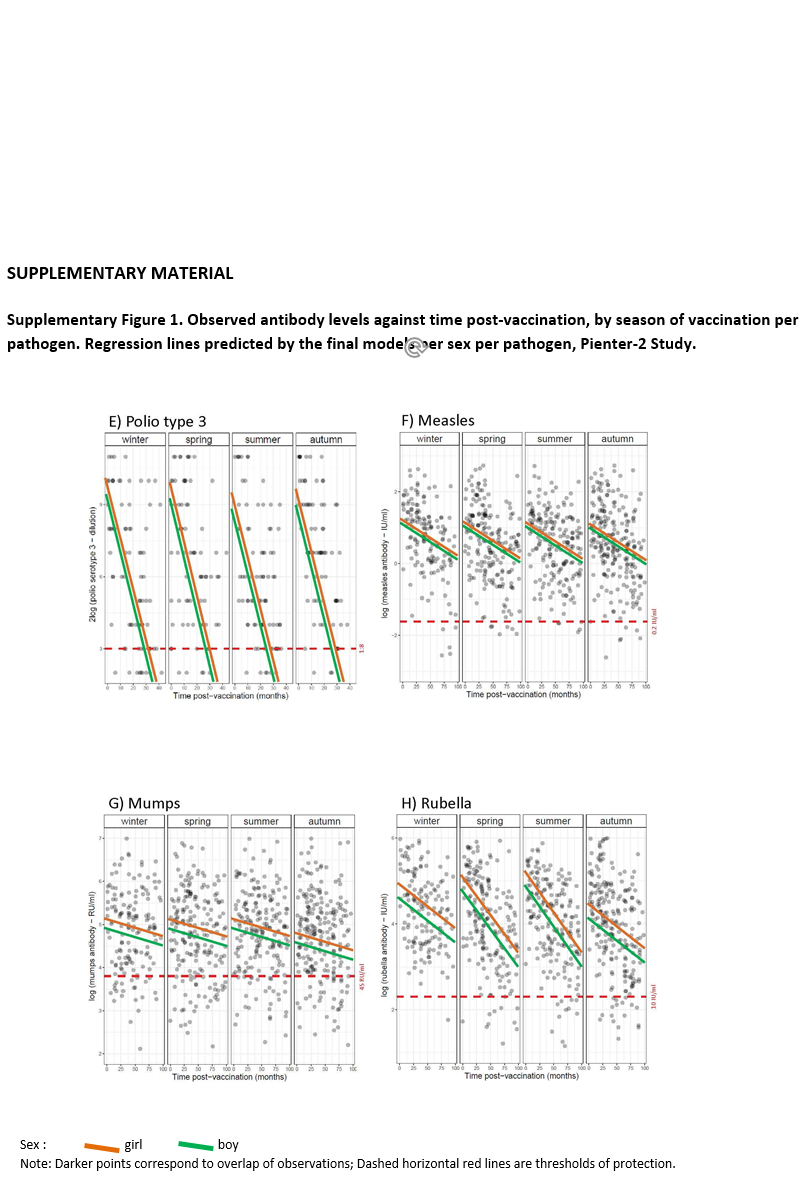

Supplement: Supplementary file 1 [file S0950268820002691sup.zip › S0950268820002691sup003.png]

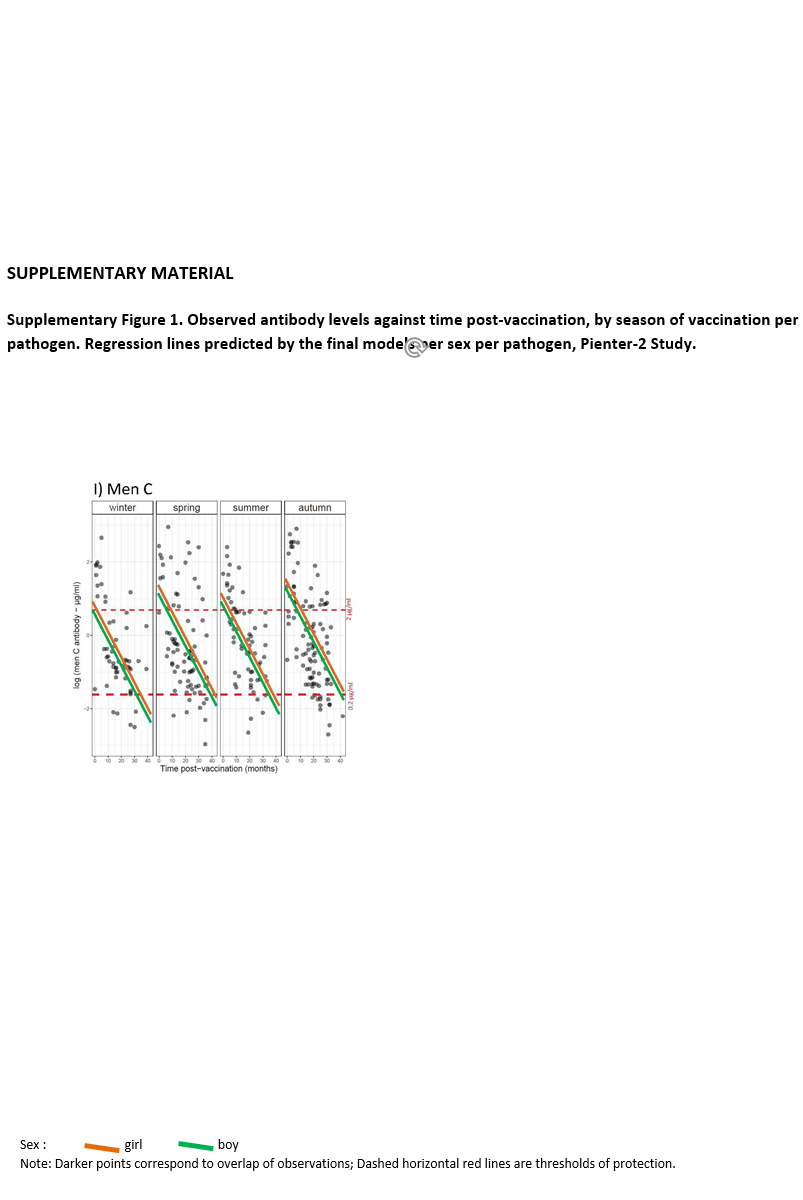

Supplement: Supplementary file 1 [file S0950268820002691sup.zip › S0950268820002691sup004.png]
